# LRP5 and LRP6 in Wnt Signaling: Similarity and Divergence

**DOI:** 10.3389/fcell.2021.670960

**Published:** 2021-05-06

**Authors:** Qian Ren, Jiongcheng Chen, Youhua Liu

**Affiliations:** ^1^State Key Laboratory of Organ Failure Research, National Clinical Research Center of Kidney Disease, Division of Nephrology, Nanfang Hospital, Southern Medical University, Guangzhou, China; ^2^Department of Pathology, University of Pittsburgh School of Medicine, Pittsburgh, PA, United States

**Keywords:** LRP5, LRP6, Wnt, β-catenin, YAP, kidney fibrosis, chronic kidney disease

## Abstract

The canonical Wnt/β-catenin signaling plays a fundamental role in regulating embryonic development, injury repair and the pathogenesis of human diseases. In vertebrates, low density lipoprotein receptor-related proteins 5 and 6 (LRP5 and LRP6), the single-pass transmembrane proteins, act as coreceptors of Wnt ligands and are indispensable for Wnt signal transduction. LRP5 and LRP6 are highly homologous and widely co-expressed in embryonic and adult tissues, and they share similar function in mediating Wnt signaling. However, they also exhibit distinct characteristics by interacting with different protein partners. As such, each of them possesses its own unique functions. In this review, we systematically discuss the similarity and divergence of LRP5 and LRP6 in mediating Wnt and other signaling in the context of kidney diseases. A better understanding of the precise role of LRP5 and LRP6 may afford us to identify and refine therapeutic targets for the treatment of a variety of human diseases.

## Introduction

Wnt/β-catenin is an evolutionarily conserved developmental signaling that plays a critical role in cell fate determination, organ development, injury repair and the pathogenesis of human diseases (Clevers, 2006; Tan et al., 2014). The term “Wnt” was derived from a combination of *Drosophila* segmental polarity gene “*Wingless*” and mouse protooncogene “*Int-1*” (Luo et al., 2007; Clevers and Nusse, 2012). Wnt ligands are a family of secreted glycoproteins, consisting of 19 members in mammals (MacDonald et al., 2009; Kawakami et al., 2013). Based on the involvement of key intracellular mediator β-catenin, Wnt signaling is divided into canonical, β-catenin-dependent and non-canonical, β-catenin-independent pathways.

In the quiescent state, β-catenin in the cytoplasm is phosphorylated and degraded by the so-called “destruction complex” in the absence of Wnt ligands. The complex consists of Axin, adenomatous polyposis coli (APC), dishevelled (Dvl), casein kinase 1 (CK1) and glycogen synthase kinase-3 (GSK-3) (Wang et al., 2018; Gajos-Michniewicz and Czyz, 2020). CK1 and GSK3 phosphorylate β-catenin sequentially, thereby tagging β-catenin for ubiquitination/degradation (Polakis, 2002; Tamai et al., 2004). In the Wnt activation state, the binding of Wnt ligands to the seven-pass transmembrane Frizzled (Fzd) receptor and its co-receptors, the low density lipoprotein receptor-related protein -5 or -6 (LRP5/6), leads to dimerization of the two receptors on cell surface and induces conformational changes of these receptors (Nusse and Clevers, 2017). The cytoplasmic tail of LRP5/6 is then phosphorylated by several protein kinases, and subsequently recruits Axin and inhibits the activity of GSK3, resulting in the dissociation of the β-catenin destruction complex (Wu et al., 2009; Kim et al., 2013). As a result, β-catenin cannot be phosphorylated and degraded. This leads to the stabilization and nuclear translocation of β-catenin, where it binds to transcription factors of the T-cell factor (TCF) and lymphoid enhancer-binding factor (LEF) families to activate the expression of Wnt target genes (van de Wetering et al., 2002; Rao and Kuhl, 2010).

In vertebrates, there are 19 different Wnt ligands and 10 Fzd receptors, but only two coreceptors (LRP5/6) in Wnt signaling (van Amerongen and Nusse, 2009). LRP5/6 are known to play a crucial role in the initiation of Wnt signal transduction, and inhibition of their function blocks Wnt/Fzd signaling (Tamai et al., 2000). While plentiful excellent reviews on Wnt signaling in human diseases, particularly the kidney disorders, have been published (Clevers and Nusse, 2012; Zhou and Liu, 2015; Zuo and Liu, 2018), relatively less is reported on the regulation and divergent functions of LRP5 and LRP6 (Joiner et al., 2013). In this study, we systematically review the structure, regulation and function of LRP5 and LRP6 in the context of kidney diseases, with emphasis on their commonality and uniqueness in mediating Wnt signaling.

## Components of Wnt Receptor Complex at the Plasma Membrane

There are several structurally unrelated, transmembrane receptor proteins that mediate different Wnt signaling (Grumolato et al., 2010). Both canonical and non-canonical Wnt ligands use common receptors of the Fzd family; however, they employ different co-receptors. LRP5/6 coreceptors are used for canonical Wnt ligands, whereas non-canonical ligands choose other transmembrane proteins (Garcia de Herreros and Dunach, 2019).

### Frizzled Receptors

In humans, there are 10 Fzd proteins, named Fzd1-10 (MacDonald and He, 2012). Fzd is a seven-pass transmembrane, atypical G protein-coupled receptor protein. The N-terminus of Fzd contains a conserved 120 amino acid cysteine-rich domain (CRD), which is the main region of Wnt binding (Nusse and Clevers, 2017). The interaction between Wnt and Fzd is promiscuous, in that a single Wnt ligand can bind multiple Fzd and a single Fzd can bind multiple Wnt ligands (Clevers, 2006). It has been shown that the specificity of Wnt signaling depends, at least in part, on the affinities between different Wnt/Fzd pairs (Hsieh, 2004). In addition, the potency of different Fzd proteins to activate canonical Wnt pathway appears not equivalent. Studies have shown that the distinction between canonical Wnt1 and non-canonical Wnt5a lies mainly in the Fzd proteins that interact with them (Holmen et al., 2002). However, it is unclear how the signal initiated by Wnt binding to the CRD of Fzd receptor is transduced across plasma membrane.

The current model for Wnt signaling is that the binding of Wnt to Fzd and LRP5/6 leads to the dimerization or clustering of these two receptors, resulting in the formation of ternary complexes together with different downstream components (Hsieh, 2004; Nusse and Clevers, 2017). Activated Fzd recruits and binds Dvl, enabling Dvl to self-polymerization via its DIX domain (Gammons et al., 2016). The Dvl DIX multimers then further recruit Axin by interacting with the Axin DIX domain (Bienz, 2014), and the locally increased Axin and its associated kinases contribute to the phosphorylation of the cytoplasmic tail of LRP6 (Tamai et al., 2004; Gerlach et al., 2018). Fzd receptors themselves, however, hardly activate the canonical Wnt pathway.

### LRP5/6 Co-Receptors

Low-density lipoprotein receptors (LDLR) are involved in a variety of cellular functions. The LDLR family members include LDL receptor, LRP (also named LRP1), megalin (LRP2), VLDL receptor, apoER2 (LRP8), SorLA/LR11, LRP1b, LRP3, MEGF7 (LRP4), and LRP5/6 (Schneider and Nimpf, 2003; Chung and Wasan, 2004). However, each member of the family is expressed in many different tissues and has a wide range of different ligands (Chung and Wasan, 2004; Joiner et al., 2013). Moreover, LRP5 and LRP6 are unique in the number and arrangement of their LDLR repeats compared to other members of the LDLR family (Brown et al., 1998). LRP5 and LRP6, as co-receptors of Wnt ligands and key components of their receptor complex, are necessary for canonical Wnt signaling (Pinson et al., 2000; Tamai et al., 2000), and are the focus of this review.

### (Pro)renin Receptor

(Pro)renin receptor (PRR) is a single-pass transmembrane protein at the plasma membrane that transmits renin and prorenin signals (Nguyen et al., 2002). It has been shown that PRR, as a component of Wnt receptor complex, can promote and augment Wnt signaling, although overexpression of PRR itself does not activate Wnt/β-catenin signaling (Cruciat et al., 2010). It is found that phosphorylation of LRP6, which is associated with LRP6 activation, requires vacuolar H^+^-adenosine triphosphatase (V-ATPase) activity. (Pro)renin receptor, as a specific adaptor between LRP6 and V-ATPase, is an essential component of the Wnt receptor complex and obligatory for its signal transduction in a (pro)renin-independent manner (Cruciat et al., 2010). Meanwhile, PRR is a direct downstream target of Wnt/β-catenin *in vitro* and *in vivo* (Li et al., 2017). As such, PRR induction and Wnt/β-catenin activation instigate a vicious, self-perpetuating cycle, leading to the amplification of the Wnt/β-catenin signaling (Li et al., 2017; Zuo and Liu, 2018).

### Other Receptors

Wnts can also transmit their signal through β-catenin-independent, non-canonical pathway. There are mainly two non-canonical Wnt signaling, the Wnt/planar cell polarity (PCP) pathway and Wnt/Ca^2+^ pathway. The Wnt/PCP signaling can be initiated through the interaction between Wnt and Fzd receptors and their co-receptors, the receptor-like tyrosine kinase (RYK) and receptor tyrosine kinase-like orphan receptor (ROR), to recruit and activate Dvl, which then serves as a scaffold and activator for RhoA and Ras. Activated RhoA and Ras then regulate the activities of Rho-associated kinase (ROCK) and c-Jun N-terminal kinase (JNK), thereby participating in polarized cell orientation and asymmetric cell movement (Kohn and Moon, 2005; Krishnamurthy and Kurzrock, 2018; VanderVorst et al., 2019). In the Wnt/Ca^2+^ pathway, Wnt binding to Fzd activates Dvl, which leads to Ca^2+^ release from the endoplasmic reticulum and thus activates Ca^2+^/calmodulin dependent kinase II (CaMKII) and calcineurin. Activated calcineurin via dephosphorylation activates the nuclear factor of activated T cells (NFAT), which translocates to the nucleus and regulates the expression of target genes, thereby regulating cell fate (Krishnamurthy and Kurzrock, 2018; Gajos-Michniewicz and Czyz, 2020).

## Structural and Functional Similarity of LRP5 and LRP6

The mature protein encoded by human and mouse *Lrp5* cDNAs are 95% identical, indicating that the protein is highly conserved during evolution (Hey et al., 1998). Similarly, the proteins encoded by mouse and human *Lrp6* share 98% identity each other (Brown et al., 1998). Meanwhile, the amino acid sequences of LRP6 and LRP5 proteins have 71% identity (Brown et al., 1998).

### Structural Domains of LRP5 and LRP6

**FIGURE 1:**
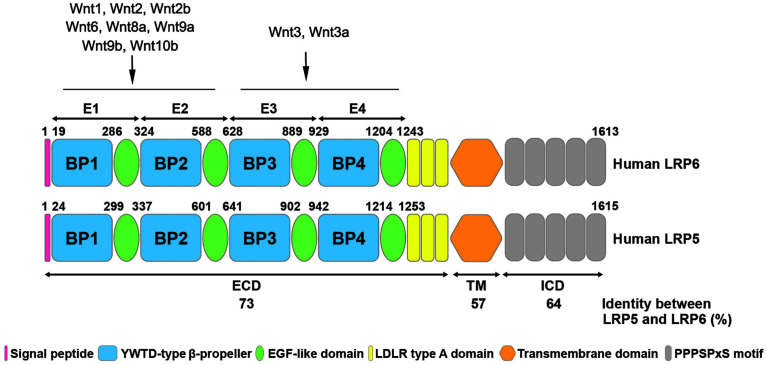
Structural similarity of human LRP5 and LRP6 proteins. The schematic diagram shows the structure of human LRP6 and LRP5 with different colors representing various domains. The numbers listed at the top of the domain represent the boundaries of signal peptides and four EGF-like domains in the full-length protein. The numbers at the bottom shows the amino acid identity between human LRP6 and LRP5. The binding sites of LRP5/6 to different Wnt ligands are indicated. ECD, extracellular domain; TM, transmembrane domain; ICD, intracellular domain.

LRP5 and LRP6 are single-pass transmembrane proteins with multiple domains (Figure 1). The first 24 amino acids at the N-terminus of LRP5 and the first 19 amino acids of LRP6 are predicted to be signal peptides for protein export across the plasma membrane (Brown et al., 1998). The mature proteins of LRP5 and LRP6 are generated by cleavage and removal of the signal peptide. The extracellular domain of LRP5 and LRP6 contains four tandem YWTD-type β-propeller (BP) domains, each followed by an epidermal growth factor (EGF)-like domain, which are named as E1 to E4 from N- to C- terminus. These extracellular domains are responsible for binding Wnt ligands and their inhibitors, such as Dickkopf-related protein 1 (DKK1) and sclerostin (He et al., 2004; Gong et al., 2010; DeBruine et al., 2017). It has been demonstrated that many Wnts such as Wnt1, Wnt2, Wnt2b, Wnt6, Wnt8a, Wnt9a, Wnt9b, and Wnt10b interact with E1E2 domain, while Wnt3 and Wnt3a prefer E3E4 domain (Figure 1). Other Wnts including Wnt7a, Wnt7b, and Wnt10a cannot be classified into either group, indicating the possibility that these Wnts may bind to regions other than E1E2 and E3E4 (Gong et al., 2010; MacDonald and He, 2012). These domains are followed by three LDLR type A domains (He et al., 2004). In general, the position and sequence of YWTD motifs are highly conserved in LRP5 and LRP6. However, the LDLR repeats between LRP6 and LRP5 are not highly conserved. Particularly, LDLR3 is only 51% identical between LRP6 and LRP5 (Brown et al., 1998). Mutagenesis studies have shown that LDLR repeats are essential for ligand binding, suggesting that LRP5 and LRP6 may bind related but not highly similar ligands. The cytoplasmic domain of LRP6 consists of 218 amino acid residues, which has 64% identity with LRP5 (Brown et al., 1998). The intracellular domains of LRP5 and LRP6 are rich in proline and serine and contain five reiterated and conserved PPPSPxS motifs, which are the binding sites for Axin and are essential for LRP6 signaling (He et al., 2004; Tamai et al., 2004) (Figure 1).

### Expression Patterns of LRP5 and LRP6 in Human Tissues

In humans, LRP5 expression level is the highest in liver, while substantial level of expression is also observed in pancreas, prostate, placenta and small intestine. LRP5 expression is detectable in ovary, thymus, skeletal muscle, colon, spleen, kidney, testis, heart, and lung as well, whereas the expression level of this receptor in brain and peripheral leukocytes is very low (Hey et al., 1998). Human LRP6 expression is highest in ovary, with significant levels in the heart, brain, placenta, lung, kidney, pancreas, spleen, and testis. Lower levels of human LRP6 are observed in liver, skeletal muscle, prostate and the mucosal lining of the colon, while the expression of LRP6 is very low in peripheral blood leukocytes, thymus and small intestine (Brown et al., 1998).

It appears that LRP6 is expressed at higher levels in the brain and kidneys than LRP5. Conversely, LRP5 appears to be expressed at higher levels than LRP6 in the liver, thymus, prostate, and small intestine (Brown et al., 1998). In addition, LRP5 is mainly expressed in renal convoluted tubules, but not in the glomeruli or collecting ducts (Figueroa et al., 2000). Both LRP5 and LRP6 are expressed in airway epithelium during lung development, whereas LRP5 but not LRP6 expression is observed in the muscular component of large blood vessels, including the aorta (Wang et al., 2005). These results indicate that LRP5 and LRP6 receptors are expressed differently in various tissues and organs, suggesting that LRP5 and LRP6 may play distinct roles in the pathogenesis of various diseases.

### LRP5 and LRP6 in Organ Development

LRP5 and LRP6 are widely co-expressed during embryonic development (Pinson et al., 2000; Houston and Wylie, 2002; He et al., 2004). *Lrp6*^–/–^ mice die at birth and exhibit a variety of severe developmental abnormalities, including a truncation of the axial skeleton, mid/hindbrain defects, limb defects, microophthalmia, and urogenital malformation (Pinson et al., 2000). These developmental defects are very similar to those of mice carrying Wnt gene mutations, especially *Wnt3a*, *Wnt1*, and *Wnt7a*, but the defects in *Lrp6* mutant embryos are usually less severe than those observed by individual *Wnt* mutants (Pinson et al., 2000). *Lrp5* ^+/–^/*Lrp6* ^–/–^ and *Lrp5* ^–/–^/*Lrp6*^–/–^ embryos arrest prior to mid-gestation, indicating functional redundancy between LRP6 and LRP5 (Kelly et al., 2004). *Lrp5*^–/–^ mice have a normal morphological appearance and are viable and fertile, but exhibit osteoporosis, subtle defects in eye vasculature (Kato et al., 2002), and metabolic abnormalities (Fujino et al., 2003), suggesting that LRP6 is more important than LRP5 in embryogenesis. Indeed, LRP6 is much more potent in activating Wnt signaling in response to a Wnt ligand in 293T cells (Holmen et al., 2002).

An allelic series of compound mutants reveals the order of progressive loss of Wnt signaling and the severity of developmental abnormalities: *Lrp5*^+/–^ (normal) < *Lrp6*^+/–^ < *Lrp5*^–/–^ < *Lrp5*^+/–^/*Lrp6*^+/–^ < *Lrp5*^–/–^/*Lrp6*^+/–^ < *Lrp6*^–/–^ < *Lrp5*^+/–^/*Lrp6*^–/–^ < *Lrp5*^–/–^/*Lrp6*^–/–^, indicating that loss of *Lrp6* alleles consistently produces a more severe phenotype than loss of *Lrp5*. This difference of LRP5 and LRP6 in organ development may be contributed by their affinity with Wnt ligands or signaling efficacy (He et al., 2004; Kelly et al., 2004). Likewise, the difference in the timing, level and location of embryonic expression of LRP5/6 or their ligands and inhibitors could account for such a separation (Kang and Robling, 2014).

## Signal Transduction Mediated by LRP5 and LRP6

When Wnt binds to and activates the Fzd, leading to the recruitment of Dvl and Axin/GSK3 complex to the plasma membrane, triggering GSK3 phosphorylation of LRP5/6 PPPSP motifs (Zeng et al., 2005; Zeng et al., 2008). These series of events eventually result in dephosphorylation of β-catenin and its stabilization (MacDonald and He, 2012).

### Kinases That Phosphorylate LRP5/6

A series of amino acid motifs on the intracellular domain of LRP5/6 are phosphorylated following Wnt binding (Tamai et al., 2004), which is essential for Wnt signaling. Up to date, five different kinds of protein kinases are known to phosphorylate LRP5/6, which can be divided into two categories. One is the proline-directed kinases that phosphorylate PPPSPxS motifs, namely GSK3, protein kinase A (PKA), PFTAIRE protein kinase (Pftk) members, and G-protein coupled receptor kinase (GRK5/6). The other is the non-proline directed kinases, namely members of the CK1 family, which phosphorylate the PPPSPxS motifs, the S/T cluster that is the conserved region preceding the first PPPSPxS motif and containing a serine and threonine residues, and other N-terminal sites (Niehrs and Shen, 2010). In addition, on the basis of kinome-wide small interfering RNA (siRNA) screen and confirmative biochemical analysis, a study demonstrates that several proline-directed mitogen-activated protein kinases (MAPKs), such as p38 MAPK, extracellular signal-regulated kinase 1/2 (ERK1/2), and c-Jun N-terminal kinase (JNK), are sufficient and necessary for phosphorylation of the PPPSP motif of LRP6 (Cervenka et al., 2011). These studies suggest that cells not only recruit one dedicated LRP6-PPPSP kinase, but also select different kinases based on cell type and the external stimulus (Cervenka et al., 2011).

### Proximal Regulatory Events of LRP5/6

The receptor complex is presumed to be a key node in Wnt signal network. However, due to the lack of molecular tools to isolate and analyze endogenous Wnt-binding components, we still lack a comprehensive understanding of the formation, composition and regulation of Wnt signalosomes. There are several proteins known to regulate LRP5/6 activity either positively or negatively. Cripto-1, which is encoded by the Cryptic family 1 gene, is shown to directly bind to LRP5/6, thereby facilitating Wnt3a binding to LRP5/6. Since Cripto-1 is located in lipid rafts, it may also promote caveolin-dependent internalization of LRP5/6, thereby enhancing the canonical Wnt/β-catenin signaling (Nagaoka et al., 2013). Biglycan, a member of the small leucine-rich proteoglycan family, has been reported to enhance canonical Wnt signaling by forming a possible trimeric complex with both Wnt and LRP6 (Berendsen et al., 2011). In addition, other studies have shown that the single-span membrane protein TMEM59 interacts with Fzd and LRP6, which promotes the formation of multimeric Wnt/Fzd complex through the intramembrane interaction, and then the Wnt/Fzd/TMEM59 assemblies merge with LRP6 to form the mature Wnt signalosomes (Gerlach et al., 2018).

In addition to the molecules that positively regulate Wnt/β-catenin signaling, several proteins that negatively regulate this pathway have also been found. The secreted proteins of DKK family, especially DKK1, antagonize Wnt/β-catenin by inhibiting Wnt co-receptor LRP6 (Mao B. et al., 2001; Niehrs, 2006). DKK1 is a high-affinity ligand for LRP6 and inhibits Wnt signaling by blocking the formation of Fzd/LRP6 complex induced by Wnt (Semenov et al., 2001). It is also reported that a secreted protein, Wise, can promote or inhibit Wnt signaling in a context-dependent manner (Itasaki et al., 2003). The Wise protein not only activates the Wnt signal cascade by mimicking some effects of Wnt ligands, but also physically interacts with LRP6 and competes with Wnt8 for bind to LRP6, thus inhibiting the Wnt signaling (Itasaki et al., 2003). Sclerostin, a secreted glycoprotein involved in the regulation of bone metabolism, has been reported to antagonize Wnt signaling by binding to the extracellular domain of LRP5/6 and disrupting Wnt-induced Fzd/LRP5/6 complex formation (Semenov et al., 2005). In addition, Mesd is a specialized molecular chaperone for LRP5/6 and is a universal inhibitor of LRP5/6 ligands (Li et al., 2005; Lu et al., 2010).

### Regulation of LRP5/6/β-Catenin Signaling After Wnt Binding

Wnt ligands induce the formation of receptor protein complexes through the successive recruitment of phosphorylation-regulated factors (Vinyoles et al., 2014). It has been proposed that the phosphorylated PPPSPxS motif of LRP5/6 directly inhibits β-catenin phosphorylation by GSK3 in a sequence and phosphorylation-dependent manner, thereby stabilizing β-catenin (Wu et al., 2009). Studies also show that Wnt signaling reduces the cytoplasmic level of GSK3 by sequestering GSK into multivesicular body (MVBS), thereby extending the half-life of β-catenin and stabilizing β-catenin (Taelman et al., 2010; Vinyoles et al., 2014).

The PPPSP motif, reiterated five times in the LRP5/6 intracellular domain, is necessary and sufficient for triggering Wnt/β-catenin signaling. An LRP6 mutant lacking the intracellular domain is defective and in fact blocks Wnt signaling (Tamai et al., 2000, 2004). In contrast, LRP5/6 mutants lacking an extracellular domain, but still being anchored on the cell membrane, seem to have constitutive activity and activate Wnt/β-catenin signaling (Mao B. et al., 2001; Mao J. et al., 2001). In addition, phosphorylated PPPSPxS peptide can sufficiently activate Wnt/β-catenin signal transduction (Wu et al., 2009). Furthermore, the transfer of a single PPPSP motif to LDLR fully activates the Wnt pathway, inducing TCF/β-catenin-responsive transcription in human cells (Tamai et al., 2004).

Several studies have shown that receptor endocytosis is involved in Wnt signaling. For example, Wnt3a and DKK1 induce LRP6 to distinct internalization pathways, thereby activating or inhibiting the β-catenin signaling. Wnt3a induces the caveolin-dependent internalization of LRP6, the phosphorylation of LRP6 and the recruitment of Axin to LRP6 on the cell membrane, leading to stabilizing β-catenin, whereas DKK1 induces clathrin-dependent internalization of LRP6 and inhibits Wnt3a-induced stabilization of β-catenin (Yamamoto et al., 2008). However, the involvement of receptor endocytosis in Wnt signaling is controversial, and contradictory results have been reported regarding the role of clathrin- and caveolin-dependent receptor internalization in Wnt signal transduction (Gagliardi et al., 2008). The pharmacological and molecular tools used to block receptor endocytosis and trafficking are pleiotropic, and sometimes the non-specific effect of these manipulations complicates the interpretations of the studies (MacDonald et al., 2009).

### Activation of LRP5/6/β-Catenin Signaling by Other Extracellular Cues

Besides Wnt ligands, LRP5/6 can respond to other extracellular cues, thereby leading to β-catenin activation. The R-Spondin (Rspo) family proteins act as potent activators of Wnt/β-catenin signaling by binding to Fzd8 and LRP6 receptors (Kazanskaya et al., 2004; Nam et al., 2006; Binnerts et al., 2007). It has been shown that human Rspo1 is a high affinity ligand for LRP6, inducing GSK3-dependent phosphorylation and activation of LRP6 (Wei et al., 2007). Interestingly, some studies show that Rspo1 does not directly bind and activate LRP6 but inhibits DKK1-mediated LRP6 internalization through its interaction with Kremen, thereby regulating the Wnt signaling (Binnerts et al., 2007). The different mechanisms may be due to different cell types.

Norrin, a cystine-knot like growth factor that is unrelated to Wnt, has been shown to bind to Fzd4 and LRP5/6 to form a ternary complex, thus activating Wnt/β-catenin signaling (Ke et al., 2013; Chang et al., 2015). In addition, parathyroid hormone (PTH) forms a ternary complex with its receptor PTH1R and co-receptor LRP6, which promotes the rapid phosphorylation of LRP6, leading to the recruitment of Axin to LRP6 and stabilization of β-catenin, and the activation of PKA is crucial for the stabilization of β-catenin induced by PTH (Wan et al., 2008). The extracellular enzyme transglutaminase 2 (TG2) has been reported to bind to LRP5/6 and act as an activating ligand for the LRP5/6, which may activate β-catenin by mediating cross-linking of the LRP5/6 receptors. This finding uncovers a novel activity of TG2 as an agonist of β-catenin signaling (Deasey et al., 2013). Collectively, these studies suggest that LRP5/6 may also act as a co-receptor of other ligands to activate β-catenin signaling.

Some extracellular cues do not bind to LRP6, *per se*, but regulate the activity of kinases used to phosphorylate LRP6, thereby activating β-catenin signaling. For example, hepatocyte growth factor (HGF) can stimulate GSK3-dependent and Wnt-independent LRP6 phosphorylation, thereby stabilizing β-catenin and activating Wnt signaling (Koraishy et al., 2014). In addition, fibroblast growth factor 2 (FGF2) promotes the phosphorylation of LRP6 and accumulation of β-catenin in an ERK1/2-dependent manner (Cervenka et al., 2011). It is also reported that receptor tyrosine kinase (RTK) signaling mediated by FGF receptor 2 (FGFR2) and FGFR3, tropomyosin receptor kinase A (TRKA) and EGF receptor (EGFR) activates Wnt/β-catenin signaling by employing ERK to phosphorylate the PPPSP motif of LRP6 (Krejci et al., 2012).

## Unique Actions of LRP6: Beyond Wnt Signaling

Although the overall structure of LRP5 and LRP6 is very similar, it has been shown that their Wnt signal transduction capabilities are not equivalent. For example, in *Xenopus* embryos, LRP6 alone is sufficient to induce axis duplication, while LRP5 is not (Tamai et al., 2000). In addition, overexpression of LRP6 alone, but not LRP5, activates the Wnt/β-catenin signaling in HEK293T cells (MacDonald et al., 2011). On the other hand, LRP5 is essential for mechanotransduction, whereas there is no clear evidence to date that LRP6 is involved in mechanical loading-induced activation of Wnt signaling (Kang and Robling, 2014). Recent studies show that Wnt ligands exhibit a preferential use of LRP5 or LRP6 (Singh et al., 2021). Three groups of Wnt ligands are identified based on their co-receptor specificity: (1) activation of Wnt signaling only through LRP6, (2) through both LRP5 and LRP6, and (3) predominantly through LRP5 (Singh et al., 2021).

Besides divergent roles of LRP5 and LRP6 in Wnt signaling (He et al., 2020; Lim et al., 2021; Singh et al., 2021), increasing evidence suggests that LRP6 displays broad actions that goes beyond Wnt signal transduction. LRP6 can interact with multiple protein partners and acts as a coreceptor for many other extracellular cues.

### LRP6 as a Coreceptor of Various Growth Factors

Apart from its well-appreciated role in Wnt signaling, LRP6 is also involved in the regulation of multiple growth factor signaling as a co-receptor. It has been shown that LRP6 interacts closely with platelet-derived growth factor receptor β (PDGFRβ) and transforming growth factor-β (TGF-β) receptor 1 (TβR1) on the cell membrane (Ren et al., 2013). PDGF phosphorylates LRP6 and stimulates the p42/p44 MAPK and JNK to promote pericytes proliferation, while DKK1, an endogenous inhibitor of LRP6, inhibits these responses. Interestingly, pericytes lacking β-catenin also respond to PDGF, confirming that the effect stimulated by PDGF is independent of the Wnt/β-catenin signaling, even though LRP6 is activated (Ren et al., 2013). These investigators also found that both TGF-β and connective tissue growth factor (CTGF) activate LRP6 within minutes, and both stimulate the p42/p44 and JNK pathways, while DKK1 blocks activation of the p42/p44 and JNK and inhibits all migration, activation, and cytoskeletal changes in pericytes in response to TGF-β or CTGF (Ren et al., 2013).

It has also been found that the CTGF domain 4 exhibits low-affinity binding to LRP6, rapidly stimulating LRP6 phosphorylation as well as downstream effectors JNK and p42/p44 activation, which can be inhibited by DKK1 or by silencing LRP6 (Johnson et al., 2017). Moreover, LRP6 binds to several G protein-coupled receptors (GPCRs) (Wo et al., 2016; Kang, 2020). GPCR ligand, such as PTH, can not only promote the interaction between LRP6 and GPCRs and activate β-catenin signaling, but also promote the binding of LRP6 to Gα(s) βγ heterotrimer and activate Gα(s) βγ-coupled GPCR signaling, suggesting that LRP6 may play a role as a general regulator of multiple GPCRs (Wan et al., 2011; Wo et al., 2016). Moreover, another study demonstrates that LRP6 can act as a co-receptor for the PTH receptor to achieve optimal activation of PTH signaling (Pellicelli et al., 2018).

### LRP6 Links Wnt to Hippo Signaling

The Hippo pathway is an evolutionarily conserved signaling cascade that regulates organ size and tissue homeostasis by governing cell proliferation and apoptosis (Hong and Guan, 2012; Imajo et al., 2012). There are two key downstream transcriptional co-activators, yes-associated protein (YAP) and transcriptional co-activator with PDZ-binding motif (TAZ), which mediate the gene regulation and biological functions of the Hippo pathway (Hong and Guan, 2012). Activation of the Hippo pathway phosphorylates YAP/TAZ, rendering them to be sequestrated in the cytoplasm and destructed by ubiquitination-dependent proteasomal degradation (Imajo et al., 2012). Therefore, the Hippo pathway limits the availability and functionality of YAP/TAZ in the nucleus by controlling its distribution and protein levels (Hong and Guan, 2012).

It is reported that in the absence of Wnt, YAP, and TAZ are components of the β-catenin destruction complex, and YAP/TAZ are associated with the destruction complex by binding to Axin (Azzolin et al., 2014). In this regard, the destruction complex is the cytoplasmic sink of YAP/TAZ (Azzolin et al., 2014). Interestingly, the progressive dissociation of YAP/TAZ with Axin parallels with the increased association of Axin with LRP6. In essence, YAP/TAZ and LRP6 apparently compete for binding to the same domain of Axin (Azzolin et al., 2014). When cells are stimulated by Wnt or overexpressed with LRP6, LRP6 releases YAP/TAZ from the destruction complex by replacing them from Axin1, thereby inducing the expression of YAP/TAZ target genes in a YAP/TAZ-dependent manner (Azzolin et al., 2014). This model is supported by another study that DKK3 stabilizes the cell-surface levels of LRP6 by uncoupling LRP6 from the Kremen-mediated internalization machinery, resulting in concomitant activation of β-catenin and YAP/TAZ (Ferrari et al., 2019).

LRP5 mutants lacking the extracellular domain act as constitutive active forms that bind Axin and induce LEF-1 activation by destabilizing Axin and stabilizing β-catenin (Mao J. et al., 2001). Intriguingly, overexpression of full-length LRP5 alone has no effect on the canonical Wnt signaling but acts synergistically with Wnt (Mao J. et al., 2001). Consistent with this result, LRP5 is shown to be associated with Axin, and Wnt3a increases the association of LRP5/Axin (Hay et al., 2009). However, either full-length LRP5 or LRP5 mutants have no effect on YAP/TAZ activity.

**FIGURE 2:**
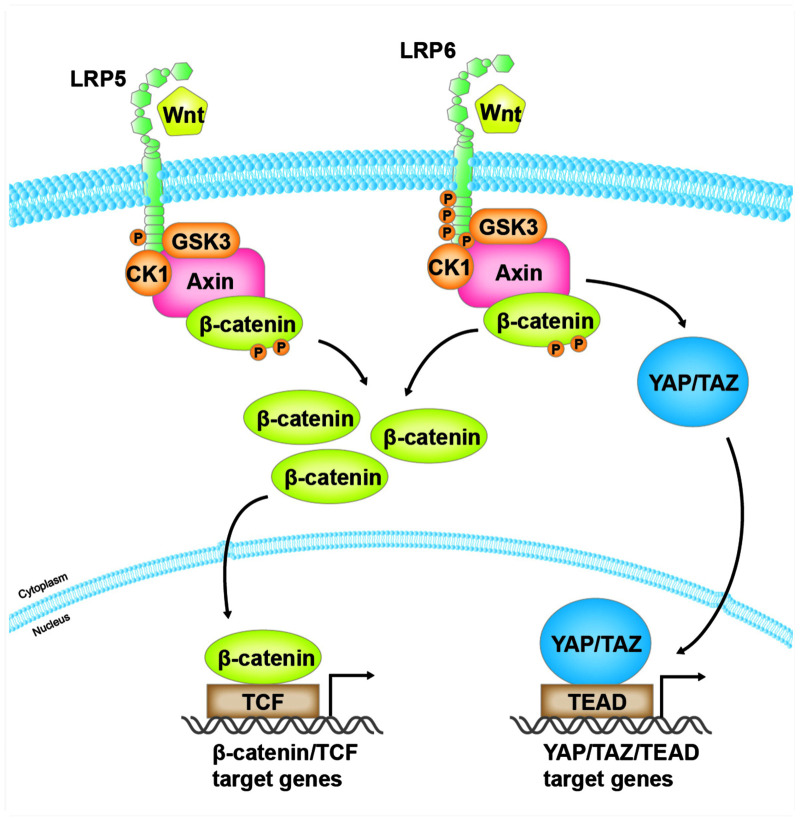
The distinct role of LRP5 and LRP6 in mediating Wnt/β-catenin and YAP/TAZ signaling. Both LRP5 and LRP6, as co-receptors of Wnt ligands, mediate Wnt/β-catenin signaling. However, when Wnt binds to and activates LRP6, activated LRP6, but not LRP5, also competitively binds to the same domain of Axin that is responsible for binding with YAP/TAZ. As a result, this leads to the release of YAP/TAZ from the destruction complex, resulting in their accumulation in the cytoplasm and translocation into the nucleus, where they bind to their cognate transcription factor TEAD and mediate the expression of their target genes.

Our own data suggest that overexpression of LRP6 alone can activate not only β-catenin but also YAP/TAZ, whereas LRP5 merely activate β-catenin (Ren et al., data unpublished). These results could be explained by two possible mechanisms. One is that overexpression of LRP6 alone leads to homo-oligomerization of LRP6 and conformational changes to form activated forms, thus recruiting Axin and resulting the activation of β-catenin and YAP/TAZ. Alternatively, due to the direct binding of LRP6 to Axin, overexpression of LRP6 alone could compete with YAP/TAZ to bind Axin, resulting in the simultaneous release of YAP/TAZ and β-catenin from the destruction complex. As shown in Figure 2, these studies may well explain how LRP6, but not LRP5, links Wnt signaling to Hippo pathway.

## Lrp5/6 and Kidney Disease

It has been shown that the *Lrp5* gene may play a role in sporadic autosomal dominant polycystic kidney disease (ADPKD) (Cnossen et al., 2016). Earlier studies have identified a total of four different LRP5 variants, which could be pathogenic predicted by *in silico* tools, suggesting that LRP5 variants may contribute to renal cystogenesis. Luciferase assays show that three of the LRP5 variants significantly reduce the activation of Wnt/β-catenin signaling (Cnossen et al., 2016). Further studies, however, are needed to validate these findings. In addition, in *Lrp6* knockout mouse embryo (18.5 days post coitum), macroscopic small cystic kidneys are visible, suggesting a PKD phenotype (Pinson et al., 2000; Wang et al., 2016). These findings indicate that LRP6 plays a pivotal role during early renal development, and that LRP5, while not affecting early renal development, may at least partially contribute to renal cystogenesis after renal maturation by affecting Wnt/β-catenin signaling.

Wnt/β-catenin signaling is reactivated after kidney injury (He et al., 2009; Zhou et al., 2016), and sustained activation of this pathway accelerates acute kidney injury (AKI) to chronic kidney diseases (CKD) progression (Xiao et al., 2016). Although LRP5 and LRP6 are co-receptors in canonical Wnt pathway, their exact role in kidney disease is unclear. Recent studies have shown that the expression of LRP5 is upregulated in renal tubules of type 1 and type 2 diabetes and unilateral ureteral obstruction (UUO) models, and knockout of LRP5 in the kidney of UUO model down-regulates TGF-β/Smad signaling and ameliorates tubulointerstitial fibrosis without changing the Wnt/β-catenin signaling (He et al., 2020). These investigators found that LRP5 could interact with TGF-β receptor I (TβRI) and TβRII, thereby promoting the formation of TβRI/TβRII heterodimers and regulating TGF-β/Smad signaling in human renal tubule epithelial cells (He et al., 2020). Phosphorylated LRP6 has also been reported to co-immunoprecipitate with TβRI. However, DKK1 has no effect on canonical TGF-β/Smad signaling in pericytes (Ren et al., 2013). The disparity in these results may be attributable to different cell types. Further studies are needed to fully elucidate the interaction between LRP6 and TβRI.

The expression of LRP6 is also up-regulated in the streptozotocin (STZ)-induced diabetic rat kidneys (Cheng et al., 2016). In another study, 2F1, a functional-blocking monoclonal antibody against the E1E2 domain of LRP6, inhibits Wnt signaling, thereby attenuating renal inflammation, proteinuria and kidney fibrosis in a type 1 diabetes model (Zhou et al., 2012). However, how LRP6 domain-specific ligand interactions mediate different signaling remains poorly understood. In this regard, a study shows that two classes of anti-LRP6 antibodies against E1E2 and E3E4 domains can either inhibit or enhance Wnt/β-catenin signaling (Gong et al., 2010; Ettenberg et al., 2010). Among them, the anti-LRP6 antibody against the E1E2 domain can specifically inhibit Wnt signaling induced by Wnt1, whereas the anti-LRP6 E3E4 domain represses Wnt signaling triggered by Wnt3a. While antibodies that recognize these separate domains may antagonize those Wnts that bind to the same domain of LRP6, antibodies may also enhance signaling mediated by Wnts that bind to different regions, through crosslinking of LRP6 molecules (Gong et al., 2010; Joiner et al., 2013). Similarly, LRP6 antibodies can also potentiate Wnt signal transduction by inhibiting the binding of antagonists, such as DKK1 and sclerostin (Gong et al., 2010). These findings indicate that anti-LRP6 antibodies may be very useful in the treatment of diseases caused by aberrant activation of Wnt/β-catenin signaling. However, one should be careful if the type of Wnt proteins expressed is not known. Because separate binding sites for different subsets of Wnt ligands determine the inhibition or potentiation of Wnt signaling, this complexity can be exploited with antibodies to differentially manipulate Wnt signaling in specific tissues or disease states.

Besides LRP6 antibodies, DKK1, as a natural inhibitor of LRP6, can effectively inhibit pericytes activation, detachment and transition to myofibroblasts *in vivo* in response to kidney injury caused by UUO and unilateral renal ischemia-reperfusion injury (UIRI), thus alleviating renal fibrosis, capillary rarefaction and inflammation (Ren et al., 2013; Johnson et al., 2017). DKK1 also represses β-catenin activation induced by Adriamycin (ADR), TGF-β1 and angiotensin II (AngII), thus alleviating podocyte injury and proteinuria (Dai et al., 2009; Wang et al., 2011; Jiang et al., 2013).

Dysregulation of LRP5/6 not only contributes to kidney diseases but also plays a critical role in the pathogenesis of CKD complications. CKD-mineral and bone disorder (CKD-MBD), characterized by mineral metabolism disorders, vascular calcification (VC), and renal osteodystrophy, is a severe complication in patients with end-stage renal disease (ESRD) (Neven et al., 2018). Mounting clinical evidence has shown that VC is an independent predictor of morbidity and mortality in CKD and ESRD (Blacher et al., 2001; Russo et al., 2011). Elevated parathyroid hormone (PTH) level is one of the major factors associated with progression of VC in hemodialysis patients (Jean et al., 2012). Moreover, PTH is a direct or indirect cause of VC in rats (Neves et al., 2007). As a co-receptor of PTH and Wnts, LRP6 plays a central role in the pathogenesis of CKD-MBD by regulating both PTH and Wnt/β-catenin signaling.

## Conclusion and Perspectives

Over the last two decades, significant progress has been made in our understanding the role of LRP5/6 in mediating Wnt signaling and other signal pathways. It becomes clear that although LRP5 and LRP6 are highly homologous, they are expressed differentially in diverse tissues and organs throughout embryonic and adult stages and possess similar yet divergent actions. In general, LRP6 appears more potent than LRP5 in transmitting Wnt signaling. In addition, LRP6 retains many unique actions by acting as a common coreceptor for numerous extracellular cues and by coupling Wnt with Hippo signaling. In short, LRP6 possesses many unique functions that extend beyond Wnt signaling.

Despite these advances, our current knowledge on the commonality and uniqueness of LRP5 and LRP6 is limited. Up to date, we still know little about the structural basis that accounts for the functional divergence of LRP5 and LRP6. Furthermore, how to target LRP5 and LRP6 by exploiting their commonality and uniqueness *in vivo* for therapeutic intervention remains in its infant stage. Given the complexity of these coreceptors, there will be many obstacles to overcome before any effective remedies can be developed and used in treating various human diseases.

## Author Contributions

QR reviewed the literature and wrote the manuscript. JC prepared the figures. YL planned the study and revised the manuscript. All authors gave the final approval of the manuscript.

## Conflict of Interest

The authors declare that the research was conducted in the absence of any commercial or financial relationships that could be construed as a potential conflict of interest.
